# Recent Biotechnology Advances in Bio-Conversion of Lignin to Lipids by Bacterial Cultures

**DOI:** 10.3389/fchem.2022.894593

**Published:** 2022-04-12

**Authors:** Huan Wang, Xiaodong Peng, Hu Li, Apostolos Giannis, Chao He

**Affiliations:** ^1^ State Key Laboratory Breeding Base of Green Pesticide and Agricultural Bioengineering, Key Laboratory of Green Pesticide and Agricultural Bioengineering, Ministry of Education, State-Local Joint Laboratory for Comprehensive Utilization of Biomass, Center for R&D of Fine Chemicals, Guizhou University, Guiyang, China; ^2^ Guizhou Industry Polytechnic College, Guiyang, China; ^3^ Guizhou Institute of Products Quality Inspection and Testing, Guiyang, China; ^4^ School of Chemical and Environmental Engineering, Technical University of Crete, University Campus, Chania, Greece; ^5^ Faculty of Engineering and Natural Sciences, Tampere University, Tampere, Finland

**Keywords:** lignin, bio-conversion, microbial depolymerization, lipids, bio-funneling

## Abstract

The complexity and recalcitrance of the lignin structure is a major barrier to its efficient utilization and commercial production of high-value products. In recent years, the “bio-funneling” transformation ability of microorganisms has provided a significant opportunity for lignin conversion and integrated biorefinery. Based on the chemical structure of lignin, this mini-review introduces the recent advances of lignin depolymerization by bacterial strains and the application of microbial lignin degradation in lipids production. Furthermore, the current challenges, future trends and perspectives for microbe-based lignin conversion to lipids are discussed.

## Introduction

The energy conversion of lignocellulosic biomass can effectively alleviate the pressure of energy crisis and environmental deterioration (Li et al., 2014; Liu et al., 2015; Li et al., 2017; Zhang and Wang, 2020). However, the sustainable and profitable development of the lignocellulose-based biorefinery industry relies on the holistic utilization of all carbon components, including cellulose, hemicellulose, and lignin (Li et al., 2015; Li H. et al., 2019; Zhao et al., 2019; Garlapati et al., 2020; Li et al., 2020a; Li et al., 2020b). Lignin is a three-dimensional biopolymer composed of three different phenylpropanoid monomers randomly polymerized by C-C and C-O bonds (Supanchaiyamat et al., 2019; Ali et al., 2021; Abu-Omar et al., 2021). Because of its high carbon-oxygen ratio and rich aromatics, it is the most promising material for generating products, such as biofuels and other high-value chemicals (Wang H. et al., 2019; Wang et al., 2021). However, the lack of efficient degradation and resource utilization technologies for lignin is the main bottleneck restricting the sustainability and cost-competitiveness of lignocellulose biorefinery (Sethupathy et al., 2022). The pulp and paper industry generates approximately 50 million tons of lignin annually with ca. 98%–99% being combusted for energy generation (Zevallos Torres et al., 2020).

Considering sustainability and industrialization, lignin-based biorefinery faces many challenges (Den et al., 2018; Yaguchi et al., 2020). The heterogeneous structure of lignin leads to its insolubility and diverse aromatics after depolymerization, which increases the difficulty of industrial transformation to target products. The diversity and complexity of linkages among the monomers increase the complexity of degradation. In addition, the degradation of lignin produces toxic inhibitors that require strong tolerance for microbial population.

The effective utilization of lignin has many significant advantages (Huang et al., 2022; Yao et al., 2022; Zhou et al., 2022). Firstly, it can improve the resource utilization efficiency and profitability of lignocellulosic biomass. Secondly, selective utilization of lignin can avoid inhibition issues resulting from lignin degradation products in the fermentation process and non-productive binding of enzymes. Thirdly, appropriate utilization of lignin could mitigate the potential environmental pollution related to huge wastewater effluents from the pulp and paper industry.

**FIGURE 1:**
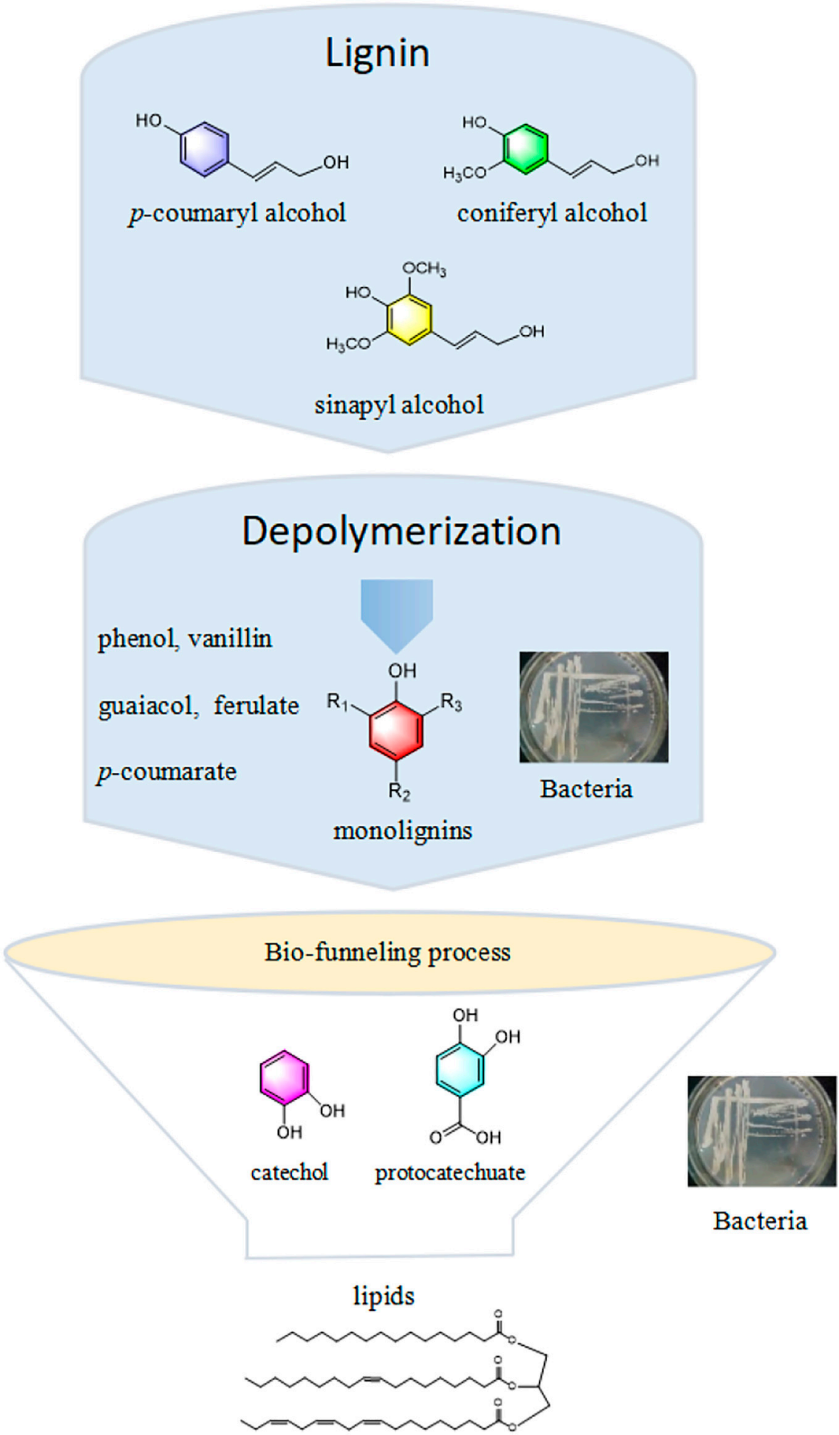
The scheme of lipids production from lignin through a bio-funneling process.

In this regard, bio-valorization is considered potentially advantageous due to its mild condition, eco-friendliness, and specificity in converting lignin into biofuels and chemicals (Singhania et al., 2022). Generally, lignin-derived bio-conversion mainly involves the microbial depolymerization of lignin into a broad spectrum of low-molecular-weight aromatics through oxidative enzymes secreted by microorganisms. These aromatics are then converted into value-added products, especially lipids, through microbial metabolism (Liu et al., 2019) (Figure 1). Conventional biodegradation achieved by fungi (e.g., brown- or white-rot fungi) has some drawbacks in terms of a long pretreatment period and poor environmental adaptability. In contrast, bacteria exhibit rapid reproduction, excellent adaptability to diverse environment, and easier genome editing, making them potential candidates for lignin-degrading strains in the future (Liu D. et al., 2018; Radhika et al., 2022). Therefore, the bio-funneling pathway process has attracted great attention due to its ability to overcome the heterogeneity of lignin-derivatives. The synthesis of lipids from lignin via the bio-funneling pathway using *Rhodococcus* strains has been widely reported in the literature (Li C. et al., 2019). Microorganism pool heterogeneous substrates into intermediates, such as protocatechuate or catechol, through a pathway known as the bio-funneling process. These intermediates further undergo central carbon metabolism to synthesize lipids by the cyclic cleavage, β-ketoadipate pathway (Salvachúa et al., 2015). To date, there are limited published reviews that have specifically addressed the use of bacteria for the production of lipids from lignin. This paper reviews current research advances on the conversion of lignin to lipids via biological pathways and possible strategies to improve lignin biotransformation, demonstrating an effective value chain from lignin to lipids in an eco-friendly and sustainable manner.

## Bio-Depolymerization of Lignin to Aromatic Compounds

Because of its natural aromatic skeleton, lignin has great potential for conversion into aromatics as biofuels or value-added chemicals, which plays an important role in improving carbon efficiency (Liao et al., 2020; Weng et al., 2021). Natural lignin mainly consists of three phenylpropanoid units, *p*-coumaryl alcohol (H-type unit), coniferyl alcohol (G-type unit), and sinapyl alcohol (S-type unit) (Becker and Wittmann, 2019), with a different number of methoxylation degrees on the aromatic rings (Mayr et al., 2021). The lignin monomers are conjugated by different bonds to form polymers with high resistance to chemical and biochemical depolymerization. Ether bonds, particularly β-O-4 ether linkages, predominate in lignin (Paananen et al., 2020; Szalaty et al., 2020).

Lignin depolymerization, the process of converting macro-molecular polymers into low-molecular-weight monomers or oligomers, is a key step in lignin valorization (Ragauskas et al., 2014). In nature, lignin degradation is induced by biological factors, such as fungi, bacteria, and abiotic factors (Radhika et al., 2021). Fungi are the most effective lignin-degrading microorganisms, mainly including white rot, brown rot, and soft rot, among which white rot has the strongest degradation ability (Ponnusamy et al., 2019; Salvachua et al., 2020). Nevertheless, the harsh growth conditions and complex genetic system of fungi greatly limit their application in industry. In addition to fungi, bacteria have also been reported to have the ability to degrade lignin. Although the degradation performance is not as good as that of fungi, bacteria demonstrate strong environmental adaptability (Xu et al., 2021). Screening for bacteria with strong lignin depolymerization capacity and identification of secreted enzymes are crucial for effective lignin utilization (Xu et al., 2019). The direct use of industrial enzymes for depolymerization can avoid severe and complex processes (e.g., high temperature and pressure, hazardous and expensive chemicals, and catalysts). Particularly, oxidases (i.e., laccases and peroxidases) are potential candidates for lignin depolymerization (Hamalainen et al., 2018).

### Bacteria Involved in the Depolymerization of Lignin

In the presence of oxygen or under anaerobic conditions, bacteria use lignin as a carbon source and then secrete oxidative enzymes to depolymerize lignin (Weng et al., 2021). *Rhodococcus*, *Proteobacteria*, and *pseudomonas* are the main bacteria that can degrade lignin effectively (Lee et al., 2019). Substrate sources and screening methods are key factors in obtaining bacteria with excellent lignin-degradation performance (Xu et al., 2019). Lignin-degrading bacteria are commonly found in lignin-rich environments, such as pulp and paper wastewater, eroded bamboo slips, soil, rotten wood, compost, and even termite gut (Xu R. et al., 2018). The commonly used screening method is to obtain the bacteria with ligninolytic capacity by using lignin or lignin-derivatives as the sole carbon source, and a suitable secondary screening model is then constructed to differentiate the degradability of lignin. For instance, dyes are often used to indicate the degradation activity of lignin due to its close structure to lignin and decolorization visualization (Xu Z. et al., 2018). *Pseudomonas putida NX-1*, *Klebsiella pneumoniae NX-1*, and *Ochrobactrum tritici* with lignin degradation capability were screened from the leaf molds using Kraft lignin as the sole carbon source, and then their capabilities to degrade lignin were verified by simulating dye decolorization and measuring decomposition enzyme activity efficiently (Xu Z. et al., 2018). The results showed that *Pseudomonas putida* NX-1 was the most capable for degrading lignin and was able to secrete laccase, lignin peroxidase (LiP), and Mn-peroxidase (MnP) efficiently. The researchers obtained two strains of lignin-degrading bacteria from the rainforest by the laccase activity against ABTS and their capabilities to degrade Kraft lignin and lignin model dimer guaiacylglycerol-β-guaiacyl ether with abundant lignin-linkage (Huang et al., 2013). *Bacillus amyloliquefaciens* SL-7 obtained from tobacco straw demonstrated better secretion of ligninolytic enzymes (MnP, LiP, and laccase) and achieved 28.55% of lignin degradation after 15 days with a comparable degradation rate to fungi, which could be an excellent strain for lignin degradation by overcoming the disadvantage of low bacterial activity (Mei et al., 2020).

### Lignin-Depolymerizing Bacterial Enzymes

Ligninolytic enzymes, which are crucial for lignin valorization, are mainly divided into two groups, i.e., lignin modifying enzymes (LMEs) and lignin-degrading auxiliary enzymes (LDAs) (Iram et al., 2021). LMEs, including laccase and peroxidase (e.g., lignin peroxidase, manganese peroxidase, multifunctional peroxidases, etc.), are predominant oxidases that can directly depolymerize lignin. Although LDAs cannot directly depolymerize lignin, they can assist LMEs in the degradation of lignin (Reshmy et al., 2022). Unlike fungal lignin-depolymerizing oxidases, bacterial lignin depolymerization is dominated by laccase and DyP (Dye decolorizing peroxidase) (de Gonzalo et al., 2016). More importantly, bacterial enzymes possess higher thermal stability and robustness as compared to fungal enzymes.

Most enzymes operate under mild conditions and even acidic conditions, but most technical lignin is undissolvable under acidic conditions. Finnish biotechnology company MetGen Oy designs and supplies a genetically engineered commercial enzyme MetZyme^®^ LIGNO^TM^ which can function at higher pH of 10–11 and elevated temperatures with a certain reduction in lignin molecular weight and increased solubility (Hamalainen et al., 2018). This enzyme-mediated scheme provides high value for lignin valorization commercially. Extreme enzymes and extremophiles are very attractive in the future because of their ability to adapt to harsh processes and perform well in the bio-conversion process, potentially bringing enzymatic lignin decomposition closer to industrial applications (Zhu et al., 2022). Zhu et al. (2017) isolated a salt-tolerant and basophilic strain of *Bacillus ligniniphilus* L1 from abyssal sediments, and then identified 15 aromatics during the bio-conversion of alkaline lignin, exhibiting significant lignin-degrading capability and adaptability to extreme environments.

## Bio-Conversion of Lignin to Lipids

As attractive feedstocks for biofuel production, lipids can be synthesized from lignin-based aromatic building blocks by oleaginous microorganisms with nearly 20% of lipids accumulation out of their dry cell weight (DCW) (Reshmy et al., 2022). Prominent oleaginous bacteria can exhibit excellent lipid accumulation capacities, such as *Acinetobacter calcoaceticus* (lipid accumulation up to 27%–38% of their DCW), *Rhodococcus opacus* (25%), and *Bacillus alcalophilus* (18%–24%) (Iram et al., 2021). Biocatalytic processes that integrate upstream depolymerization and bacterial aromatic metabolic pathways (bio-funneling process) can overcome the lignin inherent heterogeneity (Linger et al., 2014). *Rhodococcus* species, the oleaginous microorganisms, have efficient metabolism and tolerability of aromatics derived from lignin and their relative adaptability in genetic manipulation makes them promising for industrial applications (Xiong et al., 2016; Shields-Menard et al., 2017).

In general, the bio-conversion of lignin to lipids after depolymerization generally involves three stages (Chen and Wan, 2017; Lee et al., 2019): 1) conversion of lignin-degraded oligomers or monomers to catechol or protocatechuate via the bio-funneling pathway, 2) ring-opening pathways of the aromatic skeleton and β-ketoadipate pathway to produce acetyl-CoA, and 3) synthesis of lipids by oleaginous microorganisms under deficient conditions (e.g., nitrogen limitation). Microbially involved lignin bioconversion processes often pose a barrier to industrial applications aiming at high yield and high efficiency in lignin use (Ponnusamy et al., 2019).

Bio-conversion of lignin to lipids has been effectively explored in terms of improved technology and development of new processes, such as pretreatment, co-culture, and other promising enhancement technologies, etc. A list of bacteria strains together with innovative strategies to produce lipids is shown in Table 1.

**TABLE 1:** Biosynthesis pathways of lignin to lipids through bacteria.

Strains	Carbon source	Innovative strategies	Yield	References
*Rhodococcus opacus* DSM 1069	Kraft lignin	O_2_-pretreatment under alkaline environment	0.067 mg/ml	Wei et al. (2015)
*Rhodococcus opacus*	Corn stover	Ammonia fiber expansion-pretreated	32 mg/L	Wang Z. et al. (2019)
*Rhodococcus opacus* PD630 and *Rhodococcus jostii* RHA1 VanA^−^	Corn stover	Co-fermentation of wild and engineered bacteria	0.39 g lipid/g CDW	He et al. (2017)
*Rhodococcus pyridinivorans* CCZU-B16	Alkali lignin	Screening of new strains	0.52 g lipid/g CDW	Chong et al. (2017)
*Rhodococcus opacus* PD630	Corn stover	Multi-stage pretreatment method ALK-AHP	1.3 g/L	Le et al. (2017)
*Rhodococcus jostii* RHA1	Benzoate (Lignin-degradation products)	Nitrogen-limiting condition	55% of CDW	Amara et al. (2016)
*Rhodococcus opacus* DSM 1069	Pine organosolv pretreatment effluent	Organic solvent pretreatment	26.99 ± 2.88% of CDW	Wells et al. (2015)

### Pretreatment

Pretreatment is a crucial step in the integrated biorefinery (Zhang et al., 2021). Monitoring the lipid synthesis from lignin mediated by oleaginous microorganisms, it has been revealed that low-molecule-weight lignin-derived monomers were more favorable for lipid accumulation than macro-molecule of lignin. Therefore, integrating depolymerization processes that can reduce the molecular weight of lignin and break its inherent recalcitrance, with microbial synthesis processes could be applied to improve the efficiency of lipid accumulation (Li X. et al., 2019).

In the study using *Rhodococcus opacus* DSM 1069 to convert Kraft lignin to lipids, Wei et al. (2015) found that bacteria acted poorly when directly using Kraft lignin as substrate. After O_2_-pretreatment of Kraft lignin under an alkaline environment, the bacterium was able to use Kraft lignin and accumulate lipids with a maximum yield of 0.067 mg/ml. As compared to single pretreatment, combined pretreatment produced higher lipid concentration (12.8%–75.6%) because lignin-degrading bacteria released more monomers for use to achieve an optimal lipid yield of 1.83 g/L in fermentation. This indicates that combined pretreatment together with batch fermentation could be a promising strategy for efficient bio-conversion of lignin to lipids (Liu Z.-H. et al., 2018).

### Co-Culture

In general, most industrial biosynthesis processes prefer to use a single engineered microorganism to facilitate the production control. But in a single microbe, there is a competition for cellular resources in different metabolic pathways (Borchert et al., 2022). The application of microbial co-culture systems can reduce these catabolic limitations, improve streams towards desired chemicals, or enhance microbial resistance to toxicity (Singh et al., 2019). Although multiple microbial cultures can increase the complexity of the bio-funnelling pathway towards a single product, the selection of compatible partners with synergistic functions could be challenging in the co-culture system (Zuniga et al., 2020). Further investigations are needed to elucidate the interactions between microorganisms and the dynamics of community structure in the system. Given the complementary nature of different bacteria in lignin depolymerization and assimilation, the use of bacterial communities can significantly improve the efficiency of the biological upgrading of lignin for lipids accumulation.

Li X. et al. (2019) set up a wild and engineered *Rhodococcus* co-culture system, which presented excellent capabilities to degrade lignin and accumulate lipids. After selective depletion of glucose by *Rhodococcus* strains, nearly half of the lignin was then depolymerized into monomers for cell growth and lipid synthesis. The highest lignin degradation rate was 23.2% for the single strain fermentation and 33.6% for the three co-cultured strains.

Some bacteria were found to promote the growth of microalgae (Subashchandrabose et al., 2011). The microalgal growth-promoting bacterium *Azospirillum brasilense* increased the total intracellular lipid content after co-immobilization with three strains of microalgae (de-Bashan et al., 2002). Introducing these engineered microbial communities with enhanced capacity provides another new concept for the production of lipids from lignin.

### Other Promising Enhancement Technologies

Most of the lignin-derived aromatic compounds are inhibitory to microorganisms, resulting in reduction of the yield and tighter of lignocellulosic-based products (Singh et al., 2019). Thus, it is imperative to develop robust, tolerant and productive strains for effective biorefinery (Zhang and Bao, 2022). Comprehensive lipidomic research applying adaptive evolution using phenol-only carbon source for *Rhodococcus opacus* PD630 revealed a correlation between the strain’s lipid metabolism and phenol tolerance by affecting the constituent of mycolic acid and phospholipid membranes (Henson et al., 2018). Pelleted culture has been extensively studied for its high yield and ease of product collection, the low viscosity of the medium, and thus low energy consumption (Nair et al., 2016). Xu et al. (2022) reported for the first time the spontaneous formation of pellets by bacteria during fermentation with an alkaline treatment solution of maize stover as the carbon source, even in the absence of added chemical coagulants. It was found that the lipid content of the pellets was higher than that of the suspended biomass at low nitrogen concentrations. Moreover, this pellet form of microorganism has the potential for lipid production, suggesting a new strategy for the development of the biofuel industry. Liu et al. (2021) designed the “Plug-In Processes of Lignin” based on advanced pretreatment techniques to achieve a synergy of lignin biochemical conversion and carbohydrate production by reducing molecular weight and enhancing hydrophilic radicals to achieve profitable and sustainable biorefinery.

## Conclusion

The efficient resource utilization of lignin can significantly improve the economic feasibility of lignocellulosic biorefinery towards sustainability and circularity. Tremendous progress has been made in recent decades in lignin valorization, however, more efforts are still desired to produce high-value compounds from lignin efficiently and economically. Lignin depolymerization plays a crucial role in bioconversion. Microbially mediated lignin depolymerization has become a hot spot of research because of its low energy consumption and eco-friendliness. Lignin-degrading bacteria could be a breakthrough in commercial utilization of lignin due to strong tolerance under extreme conditions, fast reaction rate, and ease of genetic manipulation. Despite some recent breakthroughs in the biotransformation of lignin to lipids, nitrogen optimization for exogenous protein expression and lipid accumulation, and improvement of production efficiency are main challenges in future research work. To address the challenges of lignin biotransformation into lipids, the industrial production of lipids from lignin by bacteria can be achieved by introducing emerging technologies, including predominant pretreatment techniques, adaptive evolutions, and genetic engineering. In the future, synergistic pathways could be elaborated for the production of lipids from the metabolism of sugars and aromatic substances. An effective value chain for depolymerization and bioconversion of lignin to lipids should be established in order to provide promising prospects for the production of biofuels.
